# Microtubule-Associated Proteins with Regulatory Functions by Day and Pathological Potency at Night

**DOI:** 10.3390/cells9020357

**Published:** 2020-02-04

**Authors:** Judit Oláh, Attila Lehotzky, Sándor Szunyogh, Tibor Szénási, Ferenc Orosz, Judit Ovádi

**Affiliations:** Institute of Enzymology, Research Centre for Natural Sciences, Hungarian Academy of Sciences, 1117 Budapest, Hungary; olah.judit@ttk.mta.hu (J.O.); lehotzky.attila@ttk.mta.hu (A.L.); szunyogh.sandor@ttk.mta.hu (S.S.); szenasi.tibor@ttk.mta.hu (T.S.); orosz.ferenc@ttk.mta.hu (F.O.)

**Keywords:** microtubule ultrastructure, TPPP, mitosis inhibition, tubulin acetylation, cancer, Parkinson’s disease, inclusion, drug target

## Abstract

The sensing, integrating, and coordinating features of the eukaryotic cells are achieved by the complex ultrastructural arrays and multifarious functions of the cytoskeleton, including the microtubule network. Microtubules play crucial roles achieved by their decoration with proteins/enzymes as well as by posttranslational modifications. This review focuses on the Tubulin Polymerization Promoting Protein (TPPP/p25), a new microtubule associated protein, on its “regulatory functions by day and pathological functions at night”. Physiologically, the moonlighting TPPP/p25 modulates the dynamics and stability of the microtubule network by bundling microtubules and enhancing the tubulin acetylation due to the inhibition of tubulin deacetylases. The optimal endogenous TPPP/p25 level is crucial for its physiological functions, to the differentiation of oligodendrocytes, which are the major constituents of the myelin sheath. Pathologically, TPPP/p25 forms toxic oligomers/aggregates with α-synuclein in neurons and oligodendrocytes in Parkinson’s disease and Multiple System Atrophy, respectively; and their complex is a potential therapeutic drug target. TPPP/p25-derived microtubule hyperacetylation counteracts uncontrolled cell division. All these issues reveal the anti-mitotic and α-synuclein aggregation-promoting potency of TPPP/p25, consistent with the finding that Parkinson’s disease patients have reduced risk for certain cancers.

## 1. Tubulin Polymerization Promoting Protein (TPPP/p25), A New Microtubule Regulatory Protein

### 1.1. Major Characteristics of the TPPP Family

Microtubules are assembled from the highly conserved dimers of α- and β-tubulin and constitute the microtubule network, which is one of the major components of the eukaryotic cytoskeleton [1,2]. Microtubules are essential for cell polarity, cell shape, differentiation, and intracellular transport as well as for building of the mitotic spindles, which are the key structures in chromosome segregation during cell division. Microtubule systems can carry out a wide variety of functions at both physiological and pathological conditions that raises the following question: How can these evolutionarily-conserved proteins display multifarious functions? In living cells, microtubules interact with specific sets of microtubule-associated proteins (MAPs), including molecular motors, proteins regulating microtubule dynamics and stability and enzymes catalyzing metabolic and signaling pathways [3]. In fact, the specialized microtubule functions are determined to a large extent by the subset of partner proteins.

**Figure 1:**
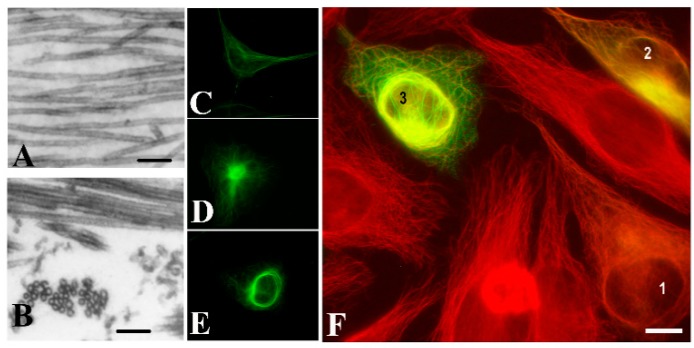
Tubulin Polymerization Promoting Protein (TPPP/p25)-derived ultrastructural organization of microtubules. Images of microtubules without or with human recombinant TPPP/p25: (**A**) control, (**B**) TPPP/p25-induced microtubule bundling, as visualized by electron microscopy. HeLa cells expressing pEGFP-TPPP/p25 (green): (**C**) control, (**D**) aggresome; (**E**) perinuclear cage; HeLa cells transiently transfected with EGFP-TPPP/p25 (green): (**F**) different expression levels produce distinct microtubule (red) ultrastructures: (**1**) alignment to the microtubule network, (**2**) aggresome formation, (**3**) perinuclear cage as visualized by immunofluorescence microscopy (selected from [9] and [8]). Scale bar: 100 nm (**A**,**B**); 10 μm (**C**–**F**).

A brain-specific protein, p25, was discovered decades ago [4,5], which was later denoted as Tubulin Polymerization Promoting Protein (TPPP/p25) and identified as a new MAP as well as a hallmark of Parkinson’s disease (PD) and other synucleinopathies [6,7]. This protein modulates the dynamics and stability of the microtubule system, aligns along the microtubule network and forms distinct microtubule ultrastructures depending on its intracellular level [8] (Figure 1).

**Figure 2:**
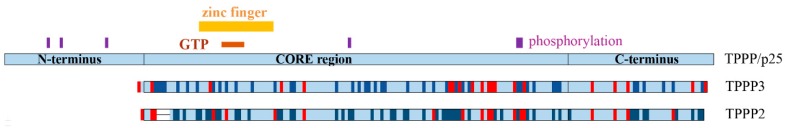
Scheme of the human TPPP/p25, TPPP3, and TPPP2. Similar and different residues are indicated by dark blue and red, respectively. The zinc finger motif, a consensus GTP-binding segment and phosphorylation sites on TPPP/p25 are also indicated.

TPPP/p25 is an intrinsically disordered protein without a well-defined 3D structure, whose middle, highly flexible CORE region is straddled by the unstructured N- and C-termini [10]. Specific binding segments in the disordered TPPP/p25 have been identified, such as a zinc finger motif, a consensus GTP-binding domain and phosphorylation sites ([11] and references therein) (Figure 2).

In vitro, TPPP/p25 is capable of inducing the polymerization of tubulin into normal, double-walled, and bundled microtubules [6]. At cellular level, TPPP/p25 co-localizes with the microtubule network in various cell lines, and its bundling activity protects the microtubules against depolymerizing agents [6,8]. The intrinsically disordered monomer TPPP/p25 is capable of dimerization stabilized by disulfide bridges [12]. The dimerization is crucial for its bundling activity; it induces the partial folding of the protein and the unstructured termini associate with other microtubules resulting in their cross-linking ([13,14] and references therein).

**Figure 3:**
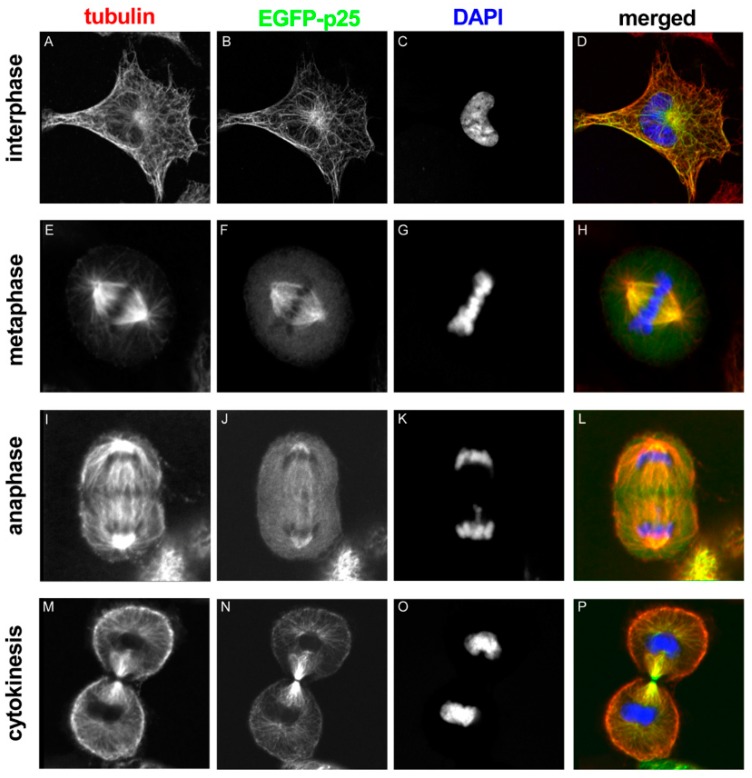
EGFP-TPPP/p25 localization in transfected NRK cells at different stages of mitosis. Tubulin, TPPP/p25 and DNA are red, green and blue in the merged images, respectively [8].

The interaction of TPPP/p25 and microtubules is very fast and dynamic, as demonstrated by fluorescence recovery after photobleaching experiments performed with normal rat kidney (NRK) cells expressing TPPP/p25 [8]. Moreover, altered localization of TPPP/p25 was detected during phases of mitosis (Figure 3). At interphase, TPPP/p25 localizes mainly on the microtubules throughout the cell: enriched TPPP/p25 can be found near to the centrosome, whereas less TPPP/p25 is present in the cell periphery. At the beginning of mitosis, before nuclear envelope breakdown, TPPP/p25 starts to detach from the microtubules. From prophase to late anaphase, free TPPP/p25 level is considerably higher in the cytoplasm. In metaphase and anaphase, TPPP/p25 accumulation is detectable only over the spindle microtubules and the centrosomes. The level of free TPPP/p25 decreases again at the telophase-cytokinesis transition and TPPP/p25 becomes preferentially enriched over the microtubules spanning the cytokinetic cleavage furrow [8]. These results indicate that TPPP/p25 binds stable interphase microtubules with high affinity (or its binding induces microtubule stabilization), whereas its association with dynamic M-phase microtubules is less efficient. The dynamic nature of the TPPP/p25 interaction with the microtubule network suggests the involvement of the protein in the reorganization of microtubule ultrastructures.

We identified two human gene sequences, which encode homologous proteins displaying approximately 60% identity with TPPP/p25; they are N-terminal-free forms denoted as TPPP2/p18 and TPPP3/p20 (cf. Figure 2) [15]. For simplicity, TPPP/p25, TPPP2 and TPPP3 terms will be used without indicating the molecular mass of the paralog forms (18 and 20 kDa).

**Figure 4:**
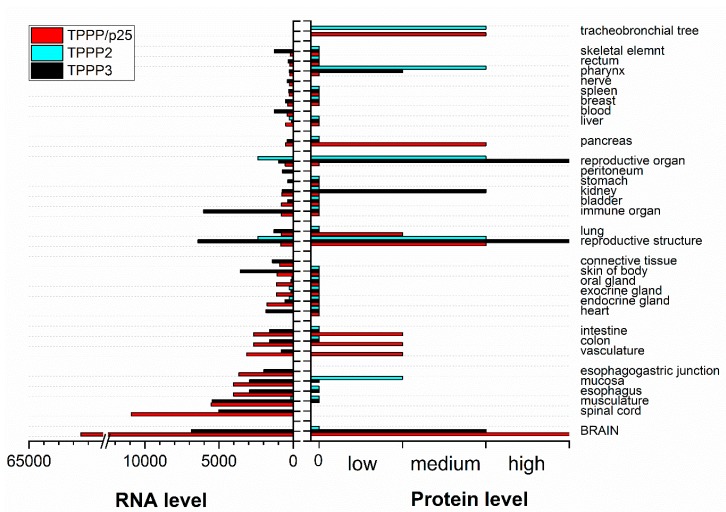
Summary of TPPP RNA and protein baseline expression data in normal human tissues based on RNA-seq expression data, Expression Atlas data and Human Protein Atlas normal tissue immunohistochemistry (https://www.targetvalidation.org) [25]. Columns without value indicate undetermined data.

Both TPPP/p25 and TPPP3, but not TPPP2, have been isolated from bovine brain, and they associate to microtubules, displaying MAP-like features [6,15] (Figure 4). TPPP/p25 has been identified at protein level predominantly in oligodendrocytes (OLGs), neuropil and fiber-like structures of the CA3 hippocampal region [5,16]. TPPP2 has not be detected in adult mammalian brain [17]. The similarity of TPPP/p25 to TPPP3 is manifested in their intrinsically disordered characteristics; in addition, they are involved in developmental processes of the brain [16,18] and the musculoskeletal system [19], respectively. TPPP2 is involved in spermatogenesis [20] and its knockdown leads to infertility in mice [21]. The main characteristics of the three TPPP proteins can be found in databases, such as the HUGO Gene Nomenclature Database: TPPP/p25 [22], TPPP3 [23], and TPPP2 [24].

### 1.2. Expression and Function of TPPP/p25 in Human Brain

TPPP/p25 is endogenously expressed in the OLGs of normal brain tissues, but not in astrocytes and neurons ([16] and references therein); in fact, its mRNA expression was found to be very low in neurons, astrocytes and microglia [26,27]. TPPP/p25 is primarily engaged in the development of projections of OLGs in the course of the differentiation that are the major constituents of the myelin ensheathing the axons [16]. The TPPP/p25 silencing by either siRNA or microRNA (miR-206) impeded the differentiation of OLGs, as we demonstrated on primary OLGs cells and in the OLG-like CG-4 cells culture [16]. The silencing posttranscriptionally reduces TPPP/p25 level and promotes the proliferation of progenitor cells, indicating the regulatory role of TPPP/p25 in the course of oligodendrogenesis [16].

Recently, Fu and co-workers [27] have identified TPPP/p25 as an OLG-enriched microtubule regulator that promotes microtubule growth from Golgi outposts and controls myelin sheath elongation, linking microtubule cytoarchitecture and myelination in the CNS. The complex cytoarchitecture of OLGs critical for myelin sheath elongation is dependent on microtubule nucleation by TPPP/p25 at Golgi outposts outside the cell body. The powerful effect of TPPP/p25 on the nucleation of the microtubules was established as well [27]. Aberrant microtubule branching, mixed microtubule polarity and shorter myelin sheaths were observed in primary OLGs of TPPP/p25 knockout mice. These mice exhibit hypomyelination with shorter, thinner myelin sheaths, which may indicate axonal transport dysfunction and degeneration. In addition, the TPPP/p25 knockout mice appeared to have breeding and motor coordination deficits [27]. Interestingly, the lack of TPPP/p25 in the embryonic brain and its presence in the postnatal brain was detected [5], coincidently with the start of myelination. These issues are in concern with our data that TPPP/p25 expression is a crucial factor of the differentiation of the OLGs [16]. Therefore, TPPP/p25 acts as a powerful nucleator of microtubules, and its propensity is important for the elongation of the microtubules for the formation of the myelin sheath by OLGs, not only for development, but also for myelin maintenance and plasticity in adult brain [27].

### 1.3. TPPP Homologs Are Extensively Wide-Spread

The vertebrate TPPPs are thought to have originated from the ancient invertebrate TPPP by two rounds of whole-genome duplication [28]. TPPP/p25 and TPPP3 are in closer phylogenetic connection with each other than either of them with TPPP2 [28]; this fact may explain their distinct functional propensities. Their microtubule binding/stabilizing function is evolutionarily conserved [29], and it is also present in invertebrates. The only TPPP of the fruit fly, *Drosophila melanogaster* [30], and that of the early branching animal, the sponge *Suberites domuncula* [29], promote microtubule bundling and polymerization potency. It was also shown that the region responsible for tubulin binding is the same in the sponge TPPP and the human TPPP/p25 proteins [29]. At lower organization level, this region is missing from TPPPs, however, a recent study in eukaryotic green alga, *Chlamydomonas reinhardtii*, demonstrated that the TPPP orthologue of the algae, the FAP265 protein, localizes at the basal bodies and in the flagella of vegetative *Chlamydomonas* cells that is essential for flagellar reassembly [31]. Since flagella or cilia are microtubule-based organelles, this finding suggests that the algal orthologue is also a microtubule-binding protein. According to bioinformatic analysis, there is a close phylogenetic connection between the presence of cilia/flagella and the occurrence of TPPP proteins [32].

Recently, the phenotypic identification and functional characterization of the Drosophila TPPP homolog named Ringmaker (Ringer; CG45057) have been reported [30]. Ringer displays a temporally dynamic expression in neurons and later in midline glia during ventral nerve cord development [30]. In fact, Ringer has been found as a major regulator of axonal microtubule organization, which is crucially required for proper axonal cytoskeletal architecture and growth during development. TPPP3 in zebrafish has been implicated in axon outgrowth as well [33,34]. Phenotypic similarities and genetic interactions with vertebrate homolog MAP1B, Futsch, have been described, indicating that both Ringer and Futsch regulate synaptic microtubule organization likely via the acetylation level of the microtubule network [35]. All these studies performed on homologs close to mammalian TPPPs suggest the role of microtubules and their associated proteins in synapse growth and organization.

TPPP/p25 localization in nerve terminals of mice and human retina has been identified; OLGs in the myelin ensheathment of optic nerve, postsynaptic nerve terminals in striations of the inner plexiform layer and a subset of amacrine cells showed immunopositivity for TPPP/p25 both in mice and human eyes [36]. The co-localization of TPPP/p25 with acetylated tubulin was detected in amacrine cells, OLG cell bodies and in synapses in the inner plexiform layer that is rich in neuropil, in which the occurrence of TPPP/p25 has been detected. This finding suggests the role of TPPP/p25 in the organization and reorganization of synaptic connections and visual integration in the eye.

### 1.4. Modulation of TPPP/p25 Expression at Transcriptional and Posttranscriptional Levels

Genome stability is involved in the coordination of mitosis and cytokinesis, where dynamic microtubules capture and faithfully segregate chromosomes into daughter cells. Very recently, high-content RNAi screen revealed multiple roles for long noncoding RNAs (lncRNAs) in cell division. For example, a robust mitotic delay was detected upon depletion of the chromatin-associated lncRNA, linc00899 [37]. The ncRNAs inhibit the translation by degradation of target RNA transcript; they have no potential to code proteins. With the development of RNA sequencing technologies and bioinformatics, it was shown that numerous ncRNAs influence expression levels via chromatin modification, transcription, and posttranscriptional processing; in addition, the abnormal expression of ncRNAs is associated with invasion, metastasis.

Extensive transcriptome analysis of *linc00899*-depleted cells suggested the interaction of *linc00899*, as lncRNA, with TPPP/p25 as a potential target that binds the genomic locus of TPPP/p25 and suppresses its transcription. In addition, the inhibition of binding of *linc00899* to TPPP/p25 resulted in the upregulation of TPPP/p25 coupled with changes in the microtubule dynamics and delay in mitosis. Therefore, the *linc0889*-dependent suppression of TPPP/p25 can control the mitotic progression, and in fact, the microtubule behavior, with functional implications beyond cell division [37]. These findings are in agreement with our observations showing the anti-mitotic effect of TPPP/p25 (see Section 2.1) and suggest pharmacological relevance, since the down-regulated *linc00899* has an anti-oncogenic effect.

### 1.5. TPPP/p25-Derived Posttranslational Modifications of the Microtubule Network

An emerging mechanism that can directly and selectively control the interactions/functions of the microtubule network is its posttranslational modification. Tubulin and microtubules are subject to a remarkable number of posttranslational modifications that have been known for many decades [38,39]. A number of enzymes involved in the catalysis of these modifications have been identified, however, understanding the roles of these modifications in determining the functions and properties of microtubules is still challenging. These modifications display considerable diversity in the intracellular milieu, which varies with development, differentiation, cell compartment, and cell cycle ([39] and references therein). The modifications affect not only the dynamics and stability of the microtubules, but also their interactions with other cellular components, which can produce specific ultrastructures, resulting in distinct physiological and pathological functions. Therefore, the understanding of cellular microtubule diversity is in the focus of recent research, including that in our laboratory.

Several known tubulin modifications have been detected on the microtubules of the cell division machinery, such as mitotic and meiotic spindles, midbody microtubules, and particularly on centrioles. The α-tubulin detyrosination, largely catalyzed by vasohibins, is involved in many microtubule-related cellular events, such as spindle formation and chromosome segregation during mitosis [40]. Deregulated detyrosination is related to cancer [41,42], linking tubulin modification, vasohibin dysfunctions and cancer [43]. Acetylation and polyglutamylation are enriched on mitotic spindle microtubules, midbody microtubules, and centrioles; however, their functional contribution is yet to be elucidated in detail. Neurons present an interesting case, as they are the only known cell type in which tubulin posttranslational modifications are strongly enriched on most cytoskeletal microtubules [44]. The neuronal microtubule cytoskeleton is involved in neuronal differentiation coupled with the accumulation of polyglutamylation, which is considered a potential regulator of microtubule properties.

The discovery of the posttranslational regulation of the microtubule network significantly contributes to our understanding of their roles in human diseases as well [39,45]. In fact, the enzymes catalyzing posttranslational modifications are accessible targets for drug development [46], and small molecule inhibitors of tubulin-modifying enzymes are promising as candidate drugs for treating serious pathologies related to aberrant tubulin modifications.

The acetylation level of microtubules and other cytosolic proteins, such as cortactin, is controlled by the opposing enzyme activities of acetyltransferases and tubulin deacetylases [18,47,48,49,50]. Cytosolic tubulin deacetylases, which specifically target the Lys-40 residue of α-tubulin, are the histone deacetylase 6 (HDAC6) and the sirtuin-2 (SIRT2) [49,50]. HDAC6 is a zinc-dependent, Class II HDAC, while SIRT2 belongs to the Class III HDACs displaying NAD+-dependent activity. Rather interestingly, while HDAC6 inhibition leads to general microtubule hyperacetylation, the hyperacetylation induced by SIRT2 inhibition is limited to perinuclear microtubules, indicating that the two enzymes might recognize specific structural context [51]. HDAC6 is ubiquitously expressed and conserved in a wide range of cell types; moreover, it is also involved in the dynein-dependent trafficking pathway as a scaffold protein, providing a connection of the dynein complex and the microtubule network [52]. TPPP/p25 inhibits the activity of HDAC6, resulting in the hyperacetylation of the microtubule network [18]. The TPPP/p25-induced HDAC6 inhibition also influences the growth velocity of the microtubule plus ends as well as the cell motility [18].

Posttranslational modifications of TPPP/p25 have been detected as well [53,54]. The phosphorylation mediated by mitogen-activated protein kinase 1 and cyclin-dependent kinase 5 (on Thr14, Ser18, and Ser160) resulted in the loss of TPPP/p25-induced assembly into intact-like microtubules [53]. Similar effect was observed upon the phosphorylation of TPPP/p25 (on the Ser residues) by LIM kinase 1 [55]. The Rho-associated coiled coil kinase also phosphorylates TPPP/p25 at Ser32, Ser107, and Ser159 without affecting its tubulin polymerization-promoting activity, but inhibiting its interaction with HDAC6 and resulting in reduced microtubule acetylation [56,57]. Therefore, the phosphorylation of distinct sites of TPPP/p25 by different kinases may play a crucial role in its multifarious functions, including its mitotic regulatory activity (cf. Figure 2).

**Figure 5:**
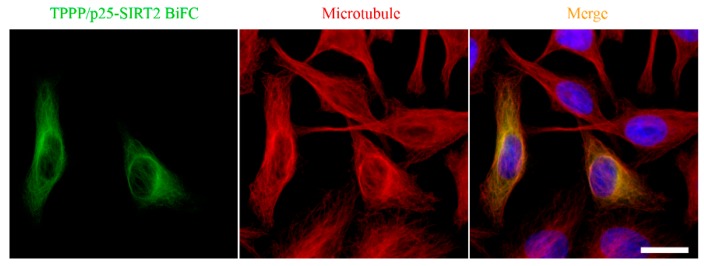
Localization of the SIRT2-TPPP/p25 complex on the microtubule network [64]. Scale bar: 10 μm.

SIRT2, as a tubulin deacetylase [50], plays an essential role in mitosis, as suggested by its increased abundance during cell division [58,59]. SIRT2 is abundant in neurons, its level is relatively high in OLGs, the major constituent of the myelin sheath [60,61,62,63]. The inhibition of SIRT2 by TPPP/p25 manifests itself within the SIRT2-tubulin-TPPP/p25 ternary complex, the concentration-dependent formation of which was quantified by experimental-based mathematical modelling [64]. Co-localization of the SIRT2-TPPP/p25 complex with the microtubule network was visualized in HeLa cells by immunofluorescence microscopy using Bimolecular Fluorescence Complementation technology [64] (Figure 5). However, the TPPP/p25-associated microtubule ultrastructures appeared to be resistant against SIRT2 activity due to the inaccessibility of the acetylated Lys40 residue. It has been proposed that the structural and functional effects of TPPP/p25 on the tubulin deacetylase SIRT2 could provide the fine-tuning of the regulation of microtubule dynamics and stability [64].

SIRT2 silencing by siRNA was reported to increase tubulin acetylation and the complexity of cellular arborization in OLGs, while its over-expression displayed opposite effects [65,66]. However, the abundance of endogenous SIRT2 expression in cultured primary OLG precursors positively correlated with increased tubulin acetylation and differentiation, indicating its counterbalancing role to prevent uncontrolled acetylation [67]. The detailed mechanism of these complex processes is largely unknown; nevertheless, the regulation of microtubule stability and dynamics through the interplay of SIRT2 and TPPP/p25 may represent an important regulatory mechanism in the cytoskeletal control either during oligodendrogenesis or in mature OLGs.

## 2. TPPP/p25 in the Focus of Cancer Research

### 2.1. Influence of TPPP/p25 on the Reorganization of the Microtubule Systems

One of our early results related to the potency of TPPP/p25 on the mitosis was obtained by a powerful in vivo method carried out on Drosophila embryos expressing GFP-tubulin fusion protein [9]. In this study, recombinant human TPPP/p25 was microinjected into the posterior pole of the cleavage embryos at the division anaphase, and the effect of TPPP/p25 on the mitosis was visualized by fluorescence confocal microscopy in the course of its diffusion [9]. TPPP/p25 inhibited mitotic spindle assembly and nuclear envelope breakdown without affecting other cellular events like centrosome replication and separation, microtubule nucleation. The specificity of the effect of TPPP/p25 on the mitosis inhibition was supported by the observation that GTP, as a regulatory ligand of the microtubule assembly, suspended the anti-mitotic effect of TPPP/p25.

The influence of TPPP/p25 on the dynamics of the microtubule system was established by monitoring the growth velocity of the microtubules by time-lapse video microscopy using the GFP coupled plus-end tracking protein EB3 in living HeLa cell model [18]. The GFP-EB3 protein was visualized parallel with TPPP/p25 in HeLa cells transiently transfected with pDsRed-TPPP/p25 construct. The analysis of the microtubule growth velocities in the TPPP/p25-expressing cells revealed that the presence of TPPP/p25 produces reduced growth velocity [18]. Additional studies showed that the effect could be attributed, at least partly, to the enhanced tubulin acetylation resulted from the inhibition of HDAC6 by TPPP/p25 [18]. The influence of TPPP/p25 on the cell motility investigated by time-lapse video microscopy using EGFP-TPPP/p25 showed that TPPP/p25 decreased the cell motility. The inhibitory effect on cell motility of TPPP/p25 overexpression was much weaker than that of paclitaxel, a microtubule stabilizing agent, but substantially stronger than that of a specific inhibitor of the tubulin deacetylase HDAC6 [18]. These findings may indicate the fine tuning of the stability of the microtubule network by TPPP/p25, which can counteract the uncontrolled cell division and display anti-cancer activity [68]. This issue is in concert with our early recognition that the expression of TPPP/p25 is negligible in cancer cell cultures, such as HeLa and many other commercial cell lines.

### 2.2. Role of TPPP/p25 and TPPP3 in Cancerous Processes

In normal brain, the microtubule network is of special importance, since it is actively involved in the maintenance of structural polarity of neurons, OLGs, and other cells, which is crucial for their physiological functions [69]. However, in the case of oligodendroglioma, a brain tumor, practically no TPPP/p25-positive cells could be detected in the brain tissue of the patients [70] (Table 1). In the case of pancreatic cancer, significantly lower TPPP/p25 expression was also found in tumor than in normal tissue [71,72]. Significantly lower TPPP/p25 expression was found also in liver tumor connected with poor prognosis [73]. The lower mRNA and/or protein expression of TPPP/p25 occurs much more frequently in the case of tumor cells in comparison to normal ones (Table 1).

A recent study on pancreatic cancer further corroborated this view; moreover, for the first time, it provided a mechanistic view on the effect of TPPP/p25. Pancreatic cancer is a highly invasive cancer with poor prognosis, the development of which is inhibited by the transcription factor, Yin Yang 1 (YY1). Now, the key role of YY1 transcription factor in the suppression of invasion and metastasis has been reported in cancer cells ([74] and references therein). ChIP-sequencing studies showed that YY1 directly binds to the promoter region of TPPP/p25 in the case of pancreatic cancer. In vivo experiments revealed that YY1 could inhibit the migration and invasion of pancreatic cancer cells by downregulating the expression of TPPP/p25 via p38/MAPK and PI3K/AKT pathways with undefined mechanism [74]. This finding indicates that TPPP/p25 is likely involved in the development of the pancreatic cancer by counteracting the inhibitory effect of YY1 manifesting in the migration, invasion and angiogenesis. Thus, TPPP/p25 has been suggested to be a novel target for the treatment of pancreatic cancer.

Genome-wide association studies were successful in identifying genomic regions with robust evidence of associations with various cancers. One of these regions is located at chromosome 5p15.33, which was consistently shown in different studies ([75] and references therein). In parallel, it was shown that gains on chromosome region 5p15.33 are a common genetic event in some cancers: Bladder [78], lung [76], and bile duct [77]. Genes at 5p15.33 with potential candidate importance, including TPPP/p25, have been identified; e.g., the amplification of this region marks a major susceptibility locus in lung cancer, and a series of genes in this region may contribute to early stage lung tumorigenesis [76]. The importance of some of the genes of this region (*TERT*, *CLPTM1L*) were verified [87], but the relationship of TPPP/p25 to malignant tumors remains to be clarified. It was suggested that amplification of TPPP/p25 may confer a growth advantage to cancerous cells due to the abnormalities in tubulin assembly and spindle formation that are common features in malignant tumors [78]. An important question, namely, how do the copy changes correlate with mRNA expression needs to be answered. There is only one study concerning TPPP/p25, which shows lower mRNA expression in 167 lung cancer tissues [75] that was interpreted as: “The role of TPPP may be complicated, and additional studies are warranted to clarify the underlying mechanisms”.

In contrast to the issue presented in relation of TPPP/p25, higher mRNA and/or protein expression of TPPP3 was found in tumor cells in comparison to normal ones in most cases investigated up to now, for example, in various lung, colorectal and ovarian tumors and in HeLa cells [79,80,81,82,83,84]. Depletion by RNAi or by shRNA inhibited tumor cell proliferation [79,80,81,82], migration and invasion [81]; inhibited lung cancer growth in vivo [82]; and survival rate was lower in patients with high expression of TPPP3 [81]. Table 1 summarizes the relationship of TPPP levels and the cancer types; however, according our knowledge, the nature of the apparently opposite effects has not been clarified.

## 3. Role of TPPP/p25 in Neurological Disorders

### 3.1. α-Synuclein and Related Pathologies

α-synuclein (SYN) is a disordered protein with high conformational plasticity (chameleon propensity) [88,89,90,91]. Structurally, SYN comprises three regions: the N-terminal region involved in lipid binding; the highly hydrophobic central NAC region, which comprises the amyloidogenic part and serves as the building block of SYN aggregates; and the negatively charged unfolded C-terminus counteracting the aggregative potency of SYN [89,90]. The phosphorylation of Ser129 or nitration of Tyr125, Tyr133, and Tyr136 promotes the formation of SYN filaments or oligomers [92], while the phosphorylation of Ser87 and Tyr125 inhibits the in vitro fibrillation [93,94,95]. Although the physiological functions of SYN have not been described in detail, it displays crucial functions highly related to synaptic plasticity, synaptic vesicle pool maintenance, and dopamine metabolism [90,96]. The disordered SYN regulates the nucleation and dynamics of microtubules as a foldable microtubule dynamase [97]; in addition, it displays direct and indirect effects on microtubule stability in the pathogenesis of PD [98].

The overexpression of SYN leads to the formation of distinct types of assemblies, including oligomers, protofibrils, fibrils, and large Lewy bodies coupled with protein dysfunction, which may result in impaired intracellular trafficking or toxicity leading to cell death [99,100,101]. SYN has been identified as the first causative gene of familial PD, the accumulation of which causes synucleinopathies including PD, dementia with Lewy bodies and multiple system atrophy (MSA) [90,101,102,103,104].

**Figure 6:**
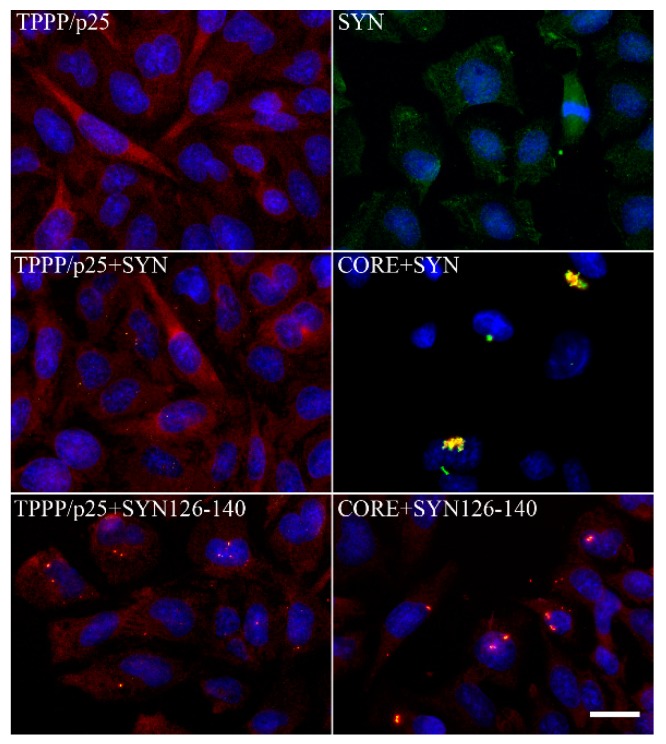
Intracellular aggregates of TPPP/p25 and α-synuclein (SYN) and their truncated forms in CHO10 cells. Uptake of the SYN and/or TPPP/p25 forms from the medium following their premixing as detected by immunofluorescence microscopy: Full length or double truncated (CORE) TPPP/p25 and/or SYN, FITC SYN126–140 (adapted from [111]). Scale bar: 5 μm.

In normal brain, the two proteins, SYN and TPPP/p25, are expressed predominantly in distinct cell types: in neurons [105,106] and in OLGs [5,16], respectively. Notwithstanding, at pathological conditions, the two hallmark proteins are co-enriched and co-localized in Lewy bodies and glial cytoplasmic inclusion in PD and MSA, respectively [7,107,108,109]. The pathological complex formation of TPPP/p25 and SYN has been characterized at molecular and cellular levels [110,111,112] (Figure 6). These data indicate that TPPP/p25 [7], besides SYN [102,103], is also a hallmark protein of synucleinopathies.

These issues have been further evolved by co-immunoprecipitation analysis carried out on HEK293T and oligodendroglial KG1C cell lines with ectopically expressed SYN and TPPP/p25. The results revealed the specific interaction of the two proteins, furthermore, the TPPP/p25-promoted oligomerization of SYN [107]. Mavroeidi and his co-workers have shown that the TPPP/p25-overexpressing oligodendroglial cells taking up human pre-formed SYN fibrils form insoluble, highly aggregated, pathological assemblies leading to the disruption of the microtubule and myelin networks [113]. Moreover, the addition of these pre-formed fibrils evoked a dramatic increase in the level of endogenous SYN in OLGs, although its mRNA level remained unchanged. The pivotal roles of both the endogenous SYN and TPPP/p25 have been proposed in the progression of aggregation and the formation of glial cytoplasmic inclusion [113]. Early relocation of TPPP/p25 from the myelin sheath, as well as from the nucleus, to the cytoplasm of OLGs was reported in MSA [108,114]. Together, these data indicate the toxic potential of the pathological SYN–TPPP/p25 assembly.

The crucial question is how do these hallmark proteins, which are normally expressed in distinct cell types, form aggregates at pathological conditions in both neurons and OLGs?

The answer lies in the progression of the disease. SYN pathology through the brain over time suggests that there should be a physical transmission of pathological SYN from one area of the brain to another ([90,91] and references therein). Evidence for cell-to-cell transmission of SYN has arisen from both in vitro and in vivo studies; neuronal cells (donors) release SYN into the extracellular space, then it is taken up by various acceptor cells [115]. Its uptake by eukaryotic cells from the media has also been reported [110]. In addition, the presence of SYN, as well as its oligomeric or the Ser129-phosphorylated forms, has also been detected in the cerebrospinal fluid of PD patients [116,117]. Lower level of total SYN and enhanced concentration of oligomeric and phosphorylated SYN have been measured, suggesting that these species may serve as diagnostic biomarkers.

The uptake of TPPP/p25 from the media by eukaryotic cells has been proved as well [110,111]. The TPPP/p25 level was analyzed in the liquor of multiple sclerosis (MS) patients. MS is a chronic inflammatory demyelinating disease and loss of TPPP/p25-positive OLGs in demyelinated lesions in the brain of MS patients has been reported [118]. The analysis of the cerebrospinal fluid of the MS patients with either clinically isolated syndromes or relapsing–remitting MS showed increased level of TPPP/p25 as compared to the non-MS patients, indicating the potency of TPPP/p25 to be a biomarker of MS [119], but further efforts are needed to apply it in the clinical practice.

### 3.2. TPPP/p25-Related Proteopathies beyond Synucleinopathies

Proteopathies, such as Alzheimer’s or Huntington’s diseases, are different than synucleinopathies (PD and MSA), but they still display common features, such as the accumulation of protein aggregates. In medicine, the term proteopathies/conformational disease refers to a class of diseases in which certain proteins become structurally abnormal, fail to fold normal conformation, lose their physiological functions and become toxic; thereby these protein species disrupt the function of cells [120,121]. The aggregation-prone disordered hallmark proteins of these diseases can coexist in the diseased brain, raising the possibility of cross-seeding of one amyloidogenic protein by aggregated states of unrelated proteins [90,122]. TPPP/p25 interacts with β-amyloid and promotes the formation of aggregates [123]. Indeed, partial co-localization of β-amyloid and TPPP/p25 has been detected in the case of diffuse Lewy body disease with Alzheimer’s disease, denoted as *mixed-type pathology* [7]. TPPP/p25 was also found to accumulate in intraneuronal granules and fibrous structures in hippocampus in the case of Alzheimer’s disease [124]. In addition, characteristic pathological protein deposits have been identified in the case of MSA and tauopathy denoted as oligodendroglial proteinopathy. Its distinct features are as follows: “More co-localization of SYN than Tau with TPPP/p25, more obvious loss of OLG density in MSA, but more prominent association of Tau protein inclusions in globular glial tauopathy” [125]. Nevertheless, the direct interaction of Tau with SYN and the partial co-localization of phospho-Tau with TPPP/p25 have been reported as well [125,126].

Tau protein is a well-characterized MAP that is tightly bound to the microtubules and stabilizes their 3D structure in eukaryotes ([127]. It is abundant in neurons, but also expressed in OLGs ([128] and references therein). TPPP/p25, as a MAP, is crucial for the decoration of the microtubule network; its expression is indispensable for the differentiation of the OLGs [16]. Nevertheless, the nature of their binding to the microtubule network is distinct: while the binding of Tau is stable, that of TPPP/p25 is highly dynamic [8]. These and additional evidence have been reported for the involvement of the microtubule system in pathological processes. Fragmentation of stable microtubules and the depletion of dynamic ones coupled with impairment of axonal transport seem to be common in the early stages of the pathology of synucleinopathies [129,130]. In PD models, earlier destabilization of the microtubule network accompanied by microtubule reorienting and the block of axonal transport were often observed. The microtubule stabilizer Epothilone D rescued microtubule defects, while the microtubule-interacting peptide davunetide improved motor functions and reduced the aggregation of SYN in mouse models of PD [129,130]. Low tubulin acetylation level, along with a concomitant impairment in axonal transport, is a common pathological hallmark in several neurodegenerative diseases [129,130]. In these pathological processes, TPPP/p25 is indirectly involved by its inhibitory potency against tubulin deacetylation [18,64]. A plausible explanation for this issue is that hyperacetylation stabilizes the microtubule network and promotes the microtubule-dependent transportation of the small toxic aggregates, including SYN, leading to the formation of aggresome at the centrosome region. Consequently, the stabilization of microtubules may be a strategy for the treatment of synucleinopathies and cancers [131,132,133].

### 3.3. Elimination of the Pathological Protein Species

The precision of cellular processes is intrinsically limited, which implies that cells naturally commit errors; in addition, cells are exposed to a multitude of stresses, both internal and environmental, which can induce molecular damage. This occurs at DNA, RNA levels arising at transcriptional or post-transcriptional processing, as well as at the protein level caused by enriched unfolded/misfolded and/or assembled proteins. Protein knockout and silencing approaches, such as CRISPR/Cas9 genome editing and RNA interference, are frequently applied for the identification of protein function and for developing therapeutic strategies. Despite these advances, most silencing systems suffer from limitations and are not always feasible. Similarly, RNA interference approaches warrant prolonged treatments and can lead to and are often associated with off-target effects ([134] and references therein). The PROteasome TArgeting Chimera (PROTAC), a recently introduced technology, can overcome some of the limitations, since it degrades proteins in a spatiotemporal manner [135,136], as we have hijacked SIRT2 as a potential anti-cancer drug target [137].

Both the microtubule-dependent autophagy and the ubiquitin–proteasome pathways, as major constituents of the cellular quality control system, play key roles in the degradation of SYN and other unwanted proteins [138]. One important aspect of SYN-mediated toxicity is the interrelation of SYN abundance and proteasome dysfunction. The degradation of TPPP/p25 by the ubiquitin–proteasome system has been reported, it is inhibited by MG132, a well-established proteasome inhibitor [8]. Blocking proteasome function impaired the ability of the centrosome to form regular microtubule asters [139]. Proteasome inhibition affects the turnover of centrosome proteins and it might increase the cytoplasmic level of these proteins, leading to ectopic nucleation of microtubules in the cytoplasm and competition with centrosomal microtubule nucleation [139].

### 3.4. Challenging Targeting of Moonlighting Proteins with Chameleon Features

The arsenal of therapies for synucleinopathies includes L-Dopa, several dopamine agonists, and inhibitors of monoamine oxidase, as well as deep brain stimulation [68,140]. Small molecules with the capacity to impede/block the assembly of toxic SYN oligomers appear to be a promising approach in the search for disease-modifying anti-PD drugs and are under development [140,141,142,143]. Nevertheless, it has to be emphasized that the extent of SYN silencing should be carefully controlled, as its near complete loss may have adverse effects on dopaminergic cell function and integrity, as revealed by the SYN knockout studies [90].

The application of drugs targeting microtubule acetylation is considered a useful strategy for therapeutic intervention [133]. Recently, growing interest has been focused on the therapeutic potency of SIRT2 inhibitors not only in cancer therapy, but also in several neurodegenerative diseases, including PD [144]. SIRT2 inhibitors prevent SYN-mediated toxicity and change the inclusion morphology in cultured neuroglioma cell line, likely due to the enhanced acetylation level of the microtubule network. In addition, their protective effects against dopaminergic cell death, both in vitro and in a Drosophila model of PD, were observed [145]. Moreover, SIRT2 inhibition was found to counteract apoptotic cell death concomitant with increased numbers of insoluble SYN-positive inclusions in a cellular model of MSA [107], suggesting the ability of SIRT2 inhibitors to rescue SYN toxicity [107,145,146,147,148]. Since endogenous SIRT2 is predominantly found in the cytoplasm of oligodendroglia rather than in that of neurons [60,61,62,63], the inhibition of SIRT2 resulting in tubulin hyperacetylation can be considered as a potential therapeutic approach in MSA. Specific HDAC6 inhibitors also exerted neuroprotection, rescued transport defects in some PD models [130,149].

**Figure 7:**
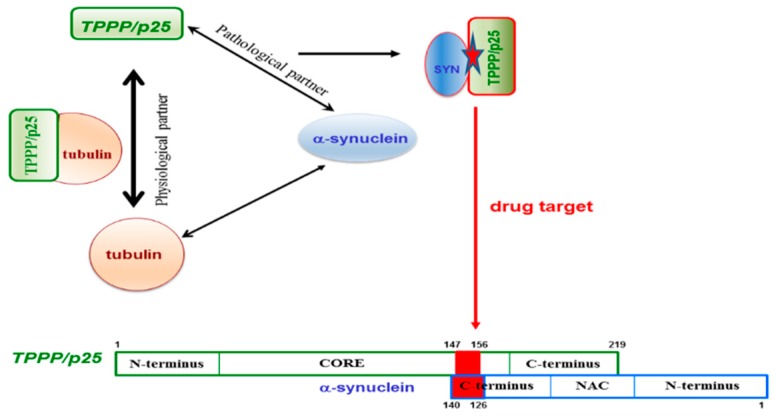
The interface of the pathological TPPP/p25–SYN complex as a specific drug target.

PD and other synucleinopathies represent an important group of neurodegenerative disorders. The hallmarks of these diseases are SYN and TPPP/p25; both proteins are disordered with chameleon characteristics and expressed distinctly in neurons and OLGs, respectively [5,16,105,106]; however, they are co-enriched and co-localized in pathological inclusions in the case of PD and MSA [7,107,108,109]. Both hallmark proteins are *neomorphic moonlighting*—in the sense that they have both physiological and pathological functions—and also have the *chameleon* features, that is, the high conformational plasticity [88,150]. Due to these unique structural and functional features, none of these hallmark proteins is an ideal drug target. However, there is an opportunity to overcome this difficulty: Targeting the SYN–TPPP/p25 complex existing exclusively at pathological conditions [110,111,112]. According to the new innovative strategy evaluated recently in our lab, the interface of the pathological complex should be targeted for the inhibition and/or destruction of TPPP/p25 and SYN assemblies [110,111,112]. This strategy offers double advantages: The uncomplexed species retain their physiological functions and the toxic side effects are diminished by the selective inhibition of the pathological complex formation (Figure 7).

To achieve the task, the binding surface of the SYN–TPPP/p25 pathological complex has to be determined. The unstructured negatively charged C-terminal segment of SYN (95–140 aa) and a segment of the highly flexible CORE region of TPPP/p25 (147–156 aa) were identified to be involved in the heterocomplex formation [110,111,112]. Nevertheless, it has to be added that the deletions of the different segments of TPPP/p25 did not result in the lack of the interaction due to its special chameleon feature; however, it was not the case with SYN. These findings expose the challenges of targeting disordered, multifunctional proteins. The opportunity to fulfil the specific drug targeting task has been achieved; the 126–140 aa segment of SYN has been identified and validated at molecular and cellular levels, as a potential anti-PD drug target [110,111,112]. All these findings reveal that, although targeting chameleon proteins is a challenging task, however, the validation of a drug target can be achieved by identifying the interface of complexes of the partner proteins existing at the given pathological conditions.

### 3.5. Are Cancer and Parkinson’s Disease Connected?

**Figure 8:**
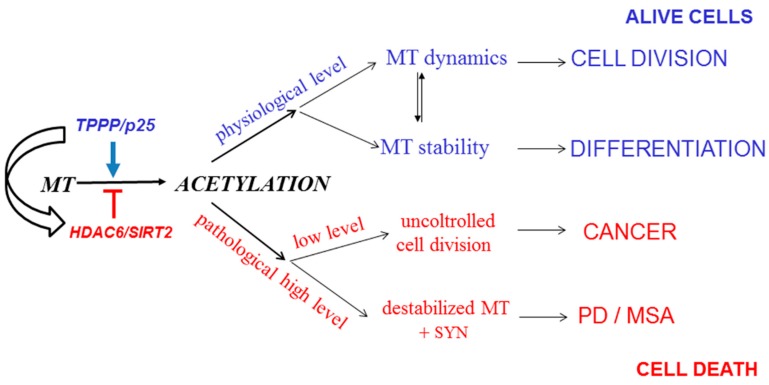
From regulation of microtubule acetylation to potential connection of Parkinson’s disease (PD) and cancer. MT: microtubule. MSA: multiple system atrophy.

A new, exciting, and emerging area is the interrelationship of cancer and neurological disorders, the two apparently distinct disease groups [151,152,153]; the cancerous tumorigenesis and the neurological disorders can be characterized by uncontrolled cell division and uncontrolled cell assemblies, respectively ([68] and references therein). However, there are observations based mostly upon population and case-control studies that suggest an inverse relationship between the etiology of certain cancers and PD. Indeed, TPPP/p25, besides its anti-proliferative activity, displays a potency to induce SYN aggregation due to its uncontrolled intracellular level [123,154]. As illustrated in Figure 8, the non-physiological level of TPPP/p25 may lead to distinct disorders, either to cancerous or to neurodegenerative diseases ([68] and references therein). In light of these findings, it is not surprising that PD patients have reduced risk for various cancers.

## Figures and Tables

**Table 1:** TPPPs in cancer.

Cancer	TPPP	Level	Level	Other	
Pancreatic	TPPP/p25	Protein	Low	High level helps invasion	[74]
Pancreatic	TPPP/p25	RNA	Low		[71]
Pancreatic	TPPP/p25	RNA	Low		[72]
Liver	TPPP/p25	RNA	Low		[73]
Oligodendroglioma	TPPP/p25	Protein	Low		[70]
Lung	TPPP/p25	RNA	Low	5p15.33	[75]
non-small cell Lung	TPPP/p25	DNA		gain of 5p15.33	[76]
Bile duct	TPPP/p25	DNA		gain of 5p15.33	[77]
Bladder	TPPP/p25	DNA		gain of 5p15.33	[78]
HeLa cells	TPPP3	RNA		Depletion by RNAi suppressed cell proliferation	[79]
Lung (Lewis carcinoma)	TPPP3	RNA		Depletion by RNAi inhibits tumor cell growth	[80]
Colorectal	TPPP3	RNA, Protein	High	Knockdown inhibited cell proliferation, migration and invasion; overall survival rate was lower in patients with high expression of TPPP3	[81]
non-small cell Lung	TPPP3	Protein	High	Knockdown by shRNA inhibited cell proliferation in vitro; depletion of TPPP3 inhibited lung cancer growth in vivo	[82]
Lung	TPPP3	Protein	High		[83]
Ovarian	TPPP3	RNA	High	10x overexpression	[84]
Clear cell sarcoma	TPPP3	RNA, Protein	High		[85]
Pancreatic ductal adenocarcinoma	TPPP3	Protein		Higher TPPP3 level indicates long survival	[86]
